# (3*Z*,3′*E*)-3,3′-[Cyclo­hexane-1,2-diylbis(aza­nylyl­idene)]bis­(indolin-2-one) *N*,*N*-dimethyl­formamide monosolvate dihydrate

**DOI:** 10.1107/S1600536812026335

**Published:** 2012-06-16

**Authors:** Shaghayegh Pezeshkpour, Hamid Khaledi, Hapipah Mohd Ali

**Affiliations:** a Department of Chemistry, University of Malaya, 50603 Kuala Lumpur, Malaysia

## Abstract

In the Schiff base mol­ecule of the title compound, C_22_H_20_N_4_O_2_·C_3_H_7_NO·2H_2_O, the cyclo­hexane ring adopts a chair conformation with the two imine groups linked at the equatorial positions. The two indolin-2-one ring systems make a dihedral angle of 65.63 (5)°. In the crystal, the Schiff base mol­ecules are connected through bifurcated N—H⋯(O,N) hydrogen bonds, forming inversion dimers. The water molecules link the dimers and the dimethylformamide molecules *via* O—H⋯O, O—H⋯N and N—H⋯O hydrogen bonds. Together with C—H⋯π and π–π [centroid–centroid distance = 3.3889 (10) Å] inter­actions a three-dimensional supra­molecular structure is formed.

## Related literature

For the structures of some Schiff bases derived from 1,2-diamino­cyclo­hexane, see: Fonseca *et al.* (2003 ▶); van den Ancker *et al.* (2006 ▶); Zhang *et al.* (2008 ▶).
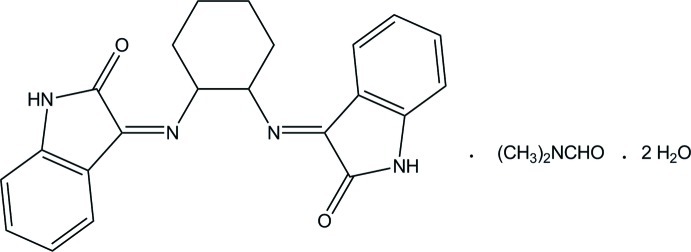


## Experimental

### 

#### Crystal data

C_22_H_20_N_4_O_2_·C_3_H_7_NO·2H_2_O
*M* *_r_* = 481.55
Triclinic, 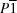

*a* = 9.1500 (9) Å
*b* = 11.3609 (12) Å
*c* = 13.6377 (14) Å
α = 109.259 (2)°
β = 108.431 (1)°
γ = 95.310 (2)°
*V* = 1238.6 (2) Å^3^
*Z* = 2
Mo *K*α radiation
μ = 0.09 mm^−1^
*T* = 100 K
0.48 × 0.42 × 0.39 mm

#### Data collection

Bruker APEXII CCD diffractometer
Absorption correction: multi-scan (*SADABS*; Sheldrick, 1996 ▶) *T*_min_ = 0.957, *T*_max_ = 0.965
6034 measured reflections
4372 independent reflections
3665 reflections with *I* > 2σ(*I*)
*R*_int_ = 0.013

#### Refinement

*R*[*F* ^2^ > 2σ(*F* ^2^)] = 0.037
*wR*(*F* ^2^) = 0.095
*S* = 1.03
4372 reflections
336 parameters
5 restraints
H atoms treated by a mixture of independent and constrained refinement
Δρ_max_ = 0.25 e Å^−3^
Δρ_min_ = −0.29 e Å^−3^

### 

Data collection: *APEX2* (Bruker, 2007 ▶); cell refinement: *SAINT* (Bruker, 2007 ▶); data reduction: *SAINT*; program(s) used to solve structure: *SHELXS97* (Sheldrick, 2008 ▶); program(s) used to refine structure: *SHELXL97* (Sheldrick, 2008 ▶); molecular graphics: *X-SEED* (Barbour, 2001 ▶); software used to prepare material for publication: *SHELXL97* and *publCIF* (Westrip, 2010 ▶).

## Supplementary Material

Crystal structure: contains datablock(s) I, global. DOI: 10.1107/S1600536812026335/is5155sup1.cif

Structure factors: contains datablock(s) I. DOI: 10.1107/S1600536812026335/is5155Isup2.hkl

Supplementary material file. DOI: 10.1107/S1600536812026335/is5155Isup3.cml

Additional supplementary materials:  crystallographic information; 3D view; checkCIF report

## Figures and Tables

**Table 1:** Hydrogen-bond geometry (Å, °) *Cg* is the centroid of the C2–C7 ring.

*D*—H⋯*A*	*D*—H	H⋯*A*	*D*⋯*A*	*D*—H⋯*A*
N1—H1*N*⋯O4	0.876 (18)	1.929 (19)	2.8036 (18)	175.6 (16)
O4—H4*A*⋯O3	0.88 (2)	1.89 (2)	2.7643 (16)	171 (2)
O5—H5*A*⋯O4	0.82 (2)	2.00 (2)	2.7901 (18)	163 (2)
O4—H4*B*⋯O5^i^	0.84 (2)	1.95 (2)	2.7415 (18)	158 (2)
N4—H4*N*⋯O1^ii^	0.872 (19)	2.222 (19)	2.9382 (18)	139.3 (16)
N4—H4*N*⋯N2^ii^	0.872 (19)	2.498 (19)	3.2246 (18)	141.3 (16)
O5—H5*B*⋯N3^iii^	0.86 (2)	1.97 (2)	2.8241 (17)	176 (2)
C10—H10*A*⋯*Cg* ^iv^	0.99	2.90	3.5799 (18)	126

Symmetry codes: (i) 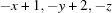; (ii) 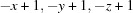; (iii) 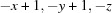; (iv) 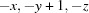.

**Fig. 1.**
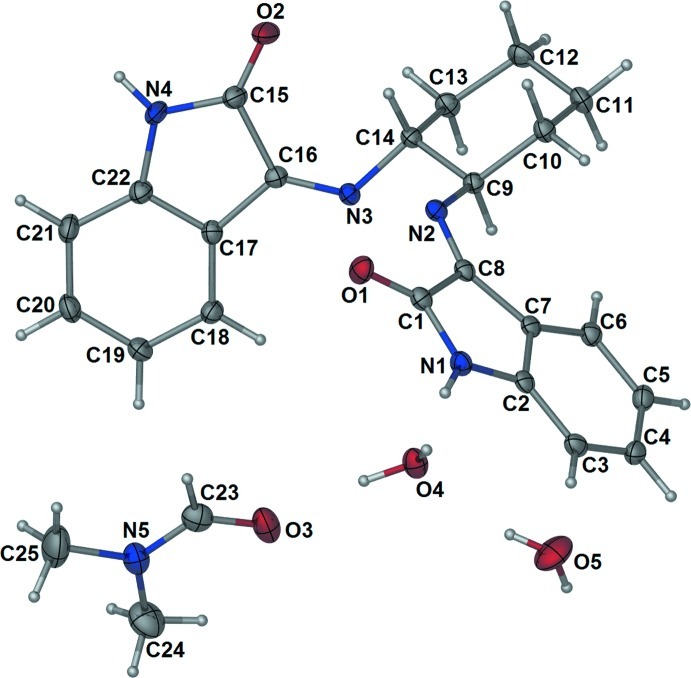
Displacement ellipsoid plot of the title compound at the 50% probability level. Hydrogen atoms are drawn as spheres of arbitrary radius.

**Fig. 2.**
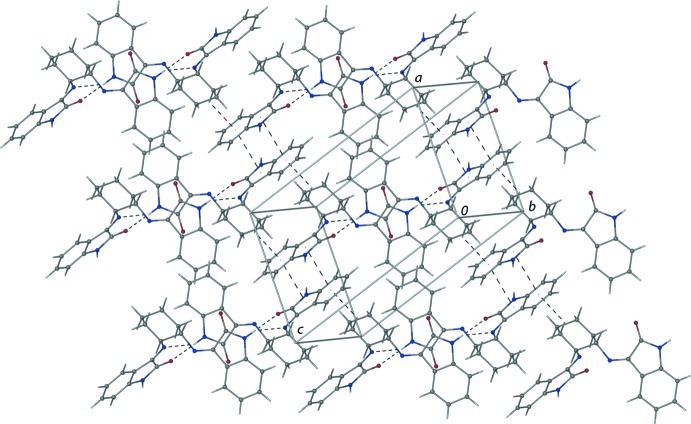
The two-dimensional-array of the Schiff molecules in the ac plane mediated by C—H···π and π···π interactions, shown as dashed lines. The solvent molecules are not shown.

## supplementary crystallographic information

### Comment

The title bis-Schiff base is the condensation product of the reaction of 1,2-diaminocyclohexane with 2 eq of isatin. The crystal structure consists of a bis-Schiff base molecule, one DMF and two water solvent molecules. As observed in similar structures (Fonseca *et al.*, 2003; van den Ancker *et al.*, 2006; Zhang *et al.*, 2008), the cyclohexane ring adopts a chair conformation with the imine links at the equatorial positions. The two isatin systems of the molecule are twisted with respect to each other by 65.63 (5)°. In the crystal, the adjacent Schiff bases are connected into a two-dimensional-array *via* C—H···π (Table 1) and π–π interactions [*Cg*1···*Cg*2^iii^ = 3.3889 (10) Å, where *Cg*1 is the centroid of N1/C1/C8/C7/C2 ring and *Cg*2^iii^ is the centroid of C2—C7 ring of the symmetry related molecule at -*x* + 1, -*y* + 1, -*z*]. The resulting network is consolidated by intermolecular N4—H···O1 and N4—H···N2 hydrogen bonding (Table 1). The solvent water molecules link the layers *via* O—H···O, O—H···N and N—H···O hydrogen bonds into a three-dimensional polymeric structure. The DMF solvent molecules are O4—H···O3 bonded to water molecules. An intramolecular C—H···O hydrogen bonding is also observed.

### Experimental

An ethanolic solution of 1,2-diaminocyclohexane (1 g, 8.76 mmol) was added slowly to a solution of isatin (3.2 g, 22 mmol) in the same solvent. The mixture was refluxed for 3 hr. The resulting yellow precipitate was filtered, washed with cold ethanol and dried over silica-gel. The title crystals were obtained from a solution of the solid in DMF.

### Refinement

The C-bound hydrogen atoms were located in the calculated positions and refined in a riding mode with C—H distances of 0.95 (*phenyl*), 0.99 (*methylene*) and 1.00 (*methine*) Å. The N-bound H atoms were found in a difference Fourier map and refined freely. The water hydrogen atoms were found in a difference Fourier map and refined with a distance restraint of O—H = 0.86 (2) Å. For all hydrogen atoms, *U*_iso_ were set to 1.2–1.5*U*_eq_(carrier atom).

### Crystal data

**Table:**

C_22_H_20_N_4_O_2_·C_3_H_7_NO·2H_2_O	*Z* = 2
*M**_r_* = 481.55	*F*(000) = 512
Triclinic, *P*1	*D*_x_ = 1.291 Mg m^−^^3^
Hall symbol: -P 1	Mo *K*α radiation, λ = 0.71073 Å
*a* = 9.1500 (9) Å	Cell parameters from 3090 reflections
*b* = 11.3609 (12) Å	θ = 2.4–30.2°
*c* = 13.6377 (14) Å	µ = 0.09 mm^−^^1^
α = 109.259 (2)°	*T* = 100 K
β = 108.431 (1)°	Block, yellow
γ = 95.310 (2)°	0.48 × 0.42 × 0.39 mm
*V* = 1238.6 (2) Å^3^	

### Data collection

**Table:**

Bruker APEXII CCD diffractometer	4372 independent reflections
Radiation source: fine-focus sealed tube	3665 reflections with *I* > 2σ(*I*)
Graphite monochromator	*R*_int_ = 0.013
φ and ω scans	θ_max_ = 25.3°, θ_min_ = 2.0°
Absorption correction: multi-scan (*SADABS*; Sheldrick, 1996)	*h* = −10→10
*T*_min_ = 0.957, *T*_max_ = 0.965	*k* = −9→13
6034 measured reflections	*l* = −16→16

### Refinement

**Table:**

Refinement on *F*^2^	Primary atom site location: structure-invariant direct methods
Least-squares matrix: full	Secondary atom site location: difference Fourier map
*R*[*F*^2^ > 2σ(*F*^2^)] = 0.037	Hydrogen site location: inferred from neighbouring sites
*wR*(*F*^2^) = 0.095	H atoms treated by a mixture of independent and constrained refinement
*S* = 1.03	*w* = 1/[σ^2^(*F*_o_^2^) + (0.0454*P*)^2^ + 0.4549*P*] where *P* = (*F*_o_^2^ + 2*F*_c_^2^)/3
4372 reflections	(Δ/σ)_max_ < 0.001
336 parameters	Δρ_max_ = 0.25 e Å^−^^3^
5 restraints	Δρ_min_ = −0.29 e Å^−^^3^

### Special details

**Table:**

Geometry. All e.s.d.'s (except the e.s.d. in the dihedral angle between two l.s. planes) are estimated using the full covariance matrix. The cell e.s.d.'s are taken into account individually in the estimation of e.s.d.'s in distances, angles and torsion angles; correlations between e.s.d.'s in cell parameters are only used when they are defined by crystal symmetry. An approximate (isotropic) treatment of cell e.s.d.'s is used for estimating e.s.d.'s involving l.s. planes.
Refinement. Refinement of *F*^2^ against ALL reflections. The weighted *R*-factor *wR* and goodness of fit *S* are based on *F*^2^, conventional *R*-factors *R* are based on *F*, with *F* set to zero for negative *F*^2^. The threshold expression of *F*^2^ > σ(*F*^2^) is used only for calculating *R*-factors(gt) *etc*. and is not relevant to the choice of reflections for refinement. *R*-factors based on *F*^2^ are statistically about twice as large as those based on *F*, and *R*- factors based on ALL data will be even larger.

### Fractional atomic coordinates and isotropic or equivalent isotropic displacement parameters (Å^2^)

**Table:**

	*x*	*y*	*z*	*U*_iso_*/*U*_eq_	
O1	0.45306 (13)	0.72380 (10)	0.27506 (8)	0.0247 (3)	
O2	0.29350 (13)	0.34365 (12)	0.46998 (9)	0.0309 (3)	
N1	0.42824 (15)	0.69114 (13)	0.09386 (10)	0.0195 (3)	
H1N	0.489 (2)	0.7633 (18)	0.1070 (14)	0.023*	
N2	0.26872 (14)	0.47022 (11)	0.18006 (10)	0.0170 (3)	
N3	0.38315 (14)	0.28305 (11)	0.25952 (10)	0.0170 (3)	
N4	0.56342 (16)	0.37451 (14)	0.55374 (11)	0.0271 (3)	
H4N	0.570 (2)	0.3877 (18)	0.6219 (16)	0.032*	
C1	0.40329 (16)	0.65863 (14)	0.17532 (12)	0.0185 (3)	
C2	0.35989 (16)	0.58948 (14)	−0.01001 (12)	0.0174 (3)	
C3	0.36705 (17)	0.58682 (15)	−0.11050 (12)	0.0214 (3)	
H3	0.4219	0.6587	−0.1152	0.026*	
C4	0.29099 (17)	0.47503 (15)	−0.20446 (12)	0.0226 (3)	
H4	0.2950	0.4703	−0.2744	0.027*	
C5	0.20937 (17)	0.37032 (15)	−0.19802 (12)	0.0220 (3)	
H5	0.1574	0.2955	−0.2636	0.026*	
C6	0.20271 (17)	0.37363 (14)	−0.09635 (12)	0.0194 (3)	
H6	0.1465	0.3019	−0.0922	0.023*	
C7	0.27986 (16)	0.48385 (14)	−0.00118 (11)	0.0169 (3)	
C8	0.30406 (16)	0.52230 (14)	0.11835 (11)	0.0168 (3)	
C9	0.17988 (16)	0.33786 (14)	0.13063 (11)	0.0165 (3)	
H9	0.2161	0.2871	0.0710	0.020*	
C10	0.00283 (17)	0.33050 (14)	0.07931 (12)	0.0203 (3)	
H10A	−0.0331	0.3830	0.1374	0.024*	
H10B	−0.0177	0.3652	0.0197	0.024*	
C11	−0.08898 (17)	0.19223 (15)	0.03049 (13)	0.0227 (3)	
H11A	−0.2034	0.1890	−0.0008	0.027*	
H11B	−0.0581	0.1411	−0.0310	0.027*	
C12	−0.05622 (18)	0.13504 (15)	0.11994 (13)	0.0243 (3)	
H12A	−0.0987	0.1799	0.1769	0.029*	
H12B	−0.1112	0.0438	0.0853	0.029*	
C13	0.12025 (17)	0.14623 (15)	0.17641 (13)	0.0220 (3)	
H13A	0.1593	0.0910	0.1217	0.026*	
H13B	0.1376	0.1153	0.2382	0.026*	
C14	0.21441 (16)	0.28386 (14)	0.22271 (12)	0.0175 (3)	
H14	0.1886	0.3385	0.2866	0.021*	
C15	0.42484 (18)	0.34589 (15)	0.46601 (12)	0.0228 (3)	
C16	0.46960 (17)	0.31155 (14)	0.36132 (12)	0.0175 (3)	
C17	0.63972 (17)	0.31781 (14)	0.40292 (12)	0.0185 (3)	
C18	0.74628 (17)	0.29560 (15)	0.34895 (12)	0.0210 (3)	
H18	0.7116	0.2681	0.2700	0.025*	
C19	0.90433 (18)	0.31444 (16)	0.41310 (13)	0.0241 (3)	
H19	0.9792	0.3002	0.3779	0.029*	
C20	0.95382 (18)	0.35400 (16)	0.52836 (13)	0.0264 (4)	
H20	1.0627	0.3664	0.5707	0.032*	
C21	0.84880 (19)	0.37610 (16)	0.58381 (12)	0.0261 (4)	
H21	0.8835	0.4028	0.6628	0.031*	
C22	0.69201 (18)	0.35752 (15)	0.51901 (12)	0.0220 (3)	
O3	0.92760 (14)	0.94186 (12)	0.26440 (10)	0.0357 (3)	
N5	1.16565 (16)	0.99322 (13)	0.40458 (11)	0.0294 (3)	
C23	1.0188 (2)	1.00428 (16)	0.36070 (14)	0.0294 (4)	
H23	0.9809	1.0655	0.4075	0.035*	
C24	1.2341 (2)	0.9040 (2)	0.33834 (18)	0.0455 (5)	
H24A	1.1535	0.8537	0.2643	0.068*	
H24B	1.2735	0.8467	0.3758	0.068*	
H24C	1.3217	0.9515	0.3302	0.068*	
C25	1.2672 (3)	1.0703 (2)	0.51971 (16)	0.0508 (5)	
H25A	1.2127	1.1326	0.5537	0.076*	
H25B	1.3651	1.1153	0.5213	0.076*	
H25C	1.2922	1.0147	0.5617	0.076*	
O4	0.62232 (14)	0.91639 (11)	0.12455 (9)	0.0262 (3)	
H4A	0.7181 (19)	0.9326 (19)	0.1742 (14)	0.039*	
H4B	0.583 (2)	0.9810 (16)	0.1350 (15)	0.039*	
O5	0.53513 (18)	0.89604 (13)	−0.09679 (11)	0.0431 (3)	
H5A	0.578 (3)	0.898 (2)	−0.0335 (14)	0.065*	
H5B	0.562 (3)	0.840 (2)	−0.1437 (17)	0.065*	

### Atomic displacement parameters (Å^2^)

**Table:**

	*U*^11^	*U*^22^	*U*^33^	*U*^12^	*U*^13^	*U*^23^
O1	0.0278 (6)	0.0218 (6)	0.0187 (6)	0.0012 (5)	0.0062 (5)	0.0038 (5)
O2	0.0242 (6)	0.0525 (8)	0.0277 (6)	0.0173 (6)	0.0155 (5)	0.0215 (6)
N1	0.0175 (6)	0.0185 (7)	0.0199 (6)	0.0003 (5)	0.0040 (5)	0.0077 (5)
N2	0.0150 (6)	0.0176 (6)	0.0191 (6)	0.0052 (5)	0.0062 (5)	0.0074 (5)
N3	0.0157 (6)	0.0177 (6)	0.0178 (6)	0.0044 (5)	0.0058 (5)	0.0070 (5)
N4	0.0245 (7)	0.0451 (9)	0.0156 (6)	0.0147 (6)	0.0098 (6)	0.0124 (6)
C1	0.0154 (7)	0.0188 (8)	0.0209 (8)	0.0052 (6)	0.0054 (6)	0.0080 (6)
C2	0.0127 (7)	0.0210 (8)	0.0195 (7)	0.0070 (6)	0.0043 (6)	0.0095 (6)
C3	0.0168 (7)	0.0269 (8)	0.0244 (8)	0.0051 (6)	0.0069 (6)	0.0151 (7)
C4	0.0199 (8)	0.0323 (9)	0.0182 (7)	0.0090 (7)	0.0067 (6)	0.0123 (7)
C5	0.0206 (8)	0.0253 (8)	0.0175 (7)	0.0071 (6)	0.0045 (6)	0.0069 (6)
C6	0.0170 (7)	0.0213 (8)	0.0196 (7)	0.0060 (6)	0.0051 (6)	0.0083 (6)
C7	0.0128 (7)	0.0211 (8)	0.0184 (7)	0.0066 (6)	0.0048 (6)	0.0096 (6)
C8	0.0131 (7)	0.0183 (8)	0.0189 (7)	0.0058 (6)	0.0053 (6)	0.0068 (6)
C9	0.0164 (7)	0.0173 (7)	0.0157 (7)	0.0044 (6)	0.0062 (6)	0.0056 (6)
C10	0.0172 (7)	0.0220 (8)	0.0211 (7)	0.0051 (6)	0.0050 (6)	0.0091 (6)
C11	0.0159 (7)	0.0241 (8)	0.0240 (8)	0.0024 (6)	0.0037 (6)	0.0083 (7)
C12	0.0199 (8)	0.0243 (8)	0.0275 (8)	0.0004 (6)	0.0084 (6)	0.0099 (7)
C13	0.0206 (8)	0.0225 (8)	0.0255 (8)	0.0041 (6)	0.0079 (6)	0.0128 (6)
C14	0.0145 (7)	0.0207 (8)	0.0184 (7)	0.0053 (6)	0.0065 (6)	0.0082 (6)
C15	0.0226 (8)	0.0299 (9)	0.0216 (8)	0.0107 (7)	0.0107 (6)	0.0132 (7)
C16	0.0194 (7)	0.0168 (7)	0.0183 (7)	0.0053 (6)	0.0081 (6)	0.0076 (6)
C17	0.0179 (7)	0.0193 (8)	0.0180 (7)	0.0050 (6)	0.0050 (6)	0.0079 (6)
C18	0.0217 (8)	0.0253 (8)	0.0178 (7)	0.0079 (6)	0.0077 (6)	0.0094 (6)
C19	0.0182 (8)	0.0308 (9)	0.0249 (8)	0.0069 (7)	0.0086 (6)	0.0115 (7)
C20	0.0179 (8)	0.0320 (9)	0.0244 (8)	0.0061 (7)	0.0022 (6)	0.0100 (7)
C21	0.0252 (8)	0.0333 (9)	0.0155 (7)	0.0085 (7)	0.0035 (6)	0.0072 (7)
C22	0.0222 (8)	0.0263 (8)	0.0188 (7)	0.0085 (7)	0.0086 (6)	0.0082 (6)
O3	0.0270 (6)	0.0351 (7)	0.0392 (7)	0.0016 (5)	0.0027 (5)	0.0176 (6)
N5	0.0266 (8)	0.0273 (8)	0.0294 (7)	0.0037 (6)	0.0050 (6)	0.0104 (6)
C23	0.0320 (9)	0.0262 (9)	0.0351 (10)	0.0067 (7)	0.0145 (8)	0.0157 (8)
C24	0.0378 (11)	0.0448 (12)	0.0528 (12)	0.0169 (9)	0.0158 (9)	0.0159 (10)
C25	0.0488 (12)	0.0529 (13)	0.0325 (10)	−0.0026 (10)	−0.0009 (9)	0.0126 (9)
O4	0.0234 (6)	0.0233 (6)	0.0272 (6)	0.0014 (5)	0.0041 (5)	0.0097 (5)
O5	0.0646 (9)	0.0387 (8)	0.0307 (7)	0.0209 (7)	0.0271 (7)	0.0071 (6)

### Geometric parameters (Å, º)

**Table:**

O1—C1	1.2195 (17)	C12—H12A	0.9900
O2—C15	1.2183 (18)	C12—H12B	0.9900
N1—C1	1.3557 (19)	C13—C14	1.530 (2)
N1—C2	1.3995 (19)	C13—H13A	0.9900
N1—H1N	0.876 (18)	C13—H13B	0.9900
N2—C8	1.2759 (19)	C14—H14	1.0000
N2—C9	1.4612 (18)	C15—C16	1.5461 (19)
N3—C16	1.2726 (18)	C16—C17	1.464 (2)
N3—C14	1.4668 (18)	C17—C18	1.390 (2)
N4—C15	1.361 (2)	C17—C22	1.396 (2)
N4—C22	1.4072 (19)	C18—C19	1.386 (2)
N4—H4N	0.872 (19)	C18—H18	0.9500
C1—C8	1.525 (2)	C19—C20	1.387 (2)
C2—C3	1.383 (2)	C19—H19	0.9500
C2—C7	1.407 (2)	C20—C21	1.392 (2)
C3—C4	1.392 (2)	C20—H20	0.9500
C3—H3	0.9500	C21—C22	1.379 (2)
C4—C5	1.388 (2)	C21—H21	0.9500
C4—H4	0.9500	O3—C23	1.228 (2)
C5—C6	1.395 (2)	N5—C23	1.323 (2)
C5—H5	0.9500	N5—C24	1.452 (2)
C6—C7	1.391 (2)	N5—C25	1.454 (2)
C6—H6	0.9500	C23—H23	0.9500
C7—C8	1.4769 (19)	C24—H24A	0.9800
C9—C10	1.5299 (19)	C24—H24B	0.9800
C9—C14	1.5318 (19)	C24—H24C	0.9800
C9—H9	1.0000	C25—H25A	0.9800
C10—C11	1.526 (2)	C25—H25B	0.9800
C10—H10A	0.9900	C25—H25C	0.9800
C10—H10B	0.9900	O4—H4A	0.880 (15)
C11—C12	1.526 (2)	O4—H4B	0.837 (15)
C11—H11A	0.9900	O5—H5A	0.821 (16)
C11—H11B	0.9900	O5—H5B	0.858 (16)
C12—C13	1.526 (2)		
			
C1—N1—C2	111.07 (13)	C12—C13—C14	112.06 (12)
C1—N1—H1N	123.4 (11)	C12—C13—H13A	109.2
C2—N1—H1N	125.1 (11)	C14—C13—H13A	109.2
C8—N2—C9	119.89 (12)	C12—C13—H13B	109.2
C16—N3—C14	123.01 (12)	C14—C13—H13B	109.2
C15—N4—C22	112.12 (12)	H13A—C13—H13B	107.9
C15—N4—H4N	124.1 (12)	N3—C14—C13	108.39 (11)
C22—N4—H4N	122.8 (12)	N3—C14—C9	106.93 (11)
O1—C1—N1	127.11 (14)	C13—C14—C9	110.37 (12)
O1—C1—C8	126.35 (13)	N3—C14—H14	110.4
N1—C1—C8	106.52 (12)	C13—C14—H14	110.4
C3—C2—N1	126.92 (14)	C9—C14—H14	110.4
C3—C2—C7	122.05 (14)	O2—C15—N4	126.70 (14)
N1—C2—C7	111.03 (12)	O2—C15—C16	127.88 (14)
C2—C3—C4	117.62 (14)	N4—C15—C16	105.37 (12)
C2—C3—H3	121.2	N3—C16—C17	125.11 (13)
C4—C3—H3	121.2	N3—C16—C15	129.81 (13)
C5—C4—C3	121.32 (14)	C17—C16—C15	105.07 (12)
C5—C4—H4	119.3	C18—C17—C22	120.24 (13)
C3—C4—H4	119.3	C18—C17—C16	132.06 (13)
C4—C5—C6	120.76 (14)	C22—C17—C16	107.67 (12)
C4—C5—H5	119.6	C19—C18—C17	118.45 (14)
C6—C5—H5	119.6	C19—C18—H18	120.8
C7—C6—C5	118.79 (14)	C17—C18—H18	120.8
C7—C6—H6	120.6	C18—C19—C20	120.38 (14)
C5—C6—H6	120.6	C18—C19—H19	119.8
C6—C7—C2	119.45 (13)	C20—C19—H19	119.8
C6—C7—C8	134.87 (14)	C19—C20—C21	121.99 (14)
C2—C7—C8	105.66 (12)	C19—C20—H20	119.0
N2—C8—C7	136.71 (14)	C21—C20—H20	119.0
N2—C8—C1	117.56 (12)	C22—C21—C20	117.02 (14)
C7—C8—C1	105.67 (12)	C22—C21—H21	121.5
N2—C9—C10	110.75 (11)	C20—C21—H21	121.5
N2—C9—C14	108.05 (11)	C21—C22—C17	121.91 (14)
C10—C9—C14	110.56 (11)	C21—C22—N4	128.43 (14)
N2—C9—H9	109.2	C17—C22—N4	109.65 (13)
C10—C9—H9	109.2	C23—N5—C24	121.01 (15)
C14—C9—H9	109.2	C23—N5—C25	122.34 (16)
C11—C10—C9	110.26 (12)	C24—N5—C25	116.64 (16)
C11—C10—H10A	109.6	O3—C23—N5	125.28 (16)
C9—C10—H10A	109.6	O3—C23—H23	117.4
C11—C10—H10B	109.6	N5—C23—H23	117.4
C9—C10—H10B	109.6	N5—C24—H24A	109.5
H10A—C10—H10B	108.1	N5—C24—H24B	109.5
C12—C11—C10	110.78 (12)	H24A—C24—H24B	109.5
C12—C11—H11A	109.5	N5—C24—H24C	109.5
C10—C11—H11A	109.5	H24A—C24—H24C	109.5
C12—C11—H11B	109.5	H24B—C24—H24C	109.5
C10—C11—H11B	109.5	N5—C25—H25A	109.5
H11A—C11—H11B	108.1	N5—C25—H25B	109.5
C13—C12—C11	111.48 (12)	H25A—C25—H25B	109.5
C13—C12—H12A	109.3	N5—C25—H25C	109.5
C11—C12—H12A	109.3	H25A—C25—H25C	109.5
C13—C12—H12B	109.3	H25B—C25—H25C	109.5
C11—C12—H12B	109.3	H4A—O4—H4B	111.8 (19)
H12A—C12—H12B	108.0	H5A—O5—H5B	111 (2)

### Hydrogen-bond geometry (Å, º)

Cg is the centroid of the C2–C7 ring.

**Table:**

*D*—H···*A*	*D*—H	H···*A*	*D*···*A*	*D*—H···*A*
N1—H1*N*···O4	0.876 (18)	1.929 (19)	2.8036 (18)	175.6 (16)
O4—H4*A*···O3	0.88 (2)	1.89 (2)	2.7643 (16)	171 (2)
O5—H5*A*···O4	0.82 (2)	2.00 (2)	2.7901 (18)	163 (2)
O4—H4*B*···O5^i^	0.84 (2)	1.95 (2)	2.7415 (18)	158 (2)
N4—H4*N*···O1^ii^	0.872 (19)	2.222 (19)	2.9382 (18)	139.3 (16)
N4—H4*N*···N2^ii^	0.872 (19)	2.498 (19)	3.2246 (18)	141.3 (16)
O5—H5*B*···N3^iii^	0.86 (2)	1.97 (2)	2.8241 (17)	176 (2)
C14—H14···O2	1.00	2.36	3.0351 (18)	124
C10—H10*A*···*Cg*^iv^	0.99	2.90	3.5799 (18)	126

Symmetry codes: (i) −*x*+1, −*y*+2, −*z*; (ii) −*x*+1, −*y*+1, −*z*+1; (iii) −*x*+1, −*y*+1, −*z*; (iv) −*x*, −*y*+1, −*z*.
